# Modulation of the Activities of Neuronal Ion Channels by Fatty Acid-Derived Pro-Resolvents

**DOI:** 10.3389/fphys.2016.00523

**Published:** 2016-11-08

**Authors:** Geunyeol Choi, Sun Wook Hwang

**Affiliations:** ^1^Department of Biomedical Sciences, Korea UniversitySeoul, South Korea; ^2^Department of Physiology, Korea University College of MedicineSeoul, South Korea

**Keywords:** resolvin, neuroprotectin, maresin, ion channel, neuron, modulation

## Abstract

Progress of inflammation depends on the balance between two biological mechanisms: pro-inflammatory and pro-resolving processes. Many extracellular and intracellular molecular components including cytokines, growth factors, steroids, neurotransmitters, and lipidergic mediators and their receptors contribute to the two processes, generated from cellular participants during inflammation. Fatty acid-derived mediators are crucial in directing the inflammatory phase and orchestrating heterogeneous reactions of participants such as inflamed cells, innate immune cells, vascular components, innervating neurons, etc. As well as activating specific types of receptor molecules, lipidergic mediators can actively control the functions of various ion channels via direct binding and/or signal transduction, thereby altering cellular functions. Lipid mediators can be divided into two classes based on which of the two processes they promote: pro-inflammatory, which includes prostaglandins and leukotrienes, and pro-resolving, which includes lipoxins, resolvins, and maresins. The research on the modulations of neuronal ion channels regarding the actions of the pro-inflammatory class has begun relatively earlier while the focus is currently expanding to cover the ion channel interaction with pro-resolvents. As a result, knowledge of inhibitory mechanisms by the pro-resolvents, historically seldom found for other known endogenous modulators or pro-inflammatory mediators, is accumulating particularly upon sensory neuronal cation channels. Diverse mechanistic explanations at molecular levels are being proposed and refined. Here we overviewed the interactions of lipidergic pro-resolvents with neuronal ion channels and outcomes from the interactions, focusing on transient receptor potential (TRP) ion channels. We also discuss unanswered hypotheses and perspectives regarding their interactions.

## Introduction

Inflammation is a protective mechanism of the body against physical/chemical injury, or infection. The inflammatory reaction is associated with functional changes in diverse tissues and cell types. In the past, the progression of inflammation from its initiation through termination was believed to be dependent only on pro-inflammatory mechanisms on the molecular and cellular levels. That is, when pro-inflammatory mediators are upregulated, the inflammatory response may last or even deteriorate because of activations of the cellular players and their signal transduction. Conversely, when those are downregulated, the inflammatory response may passively subside. This indicates that pro-inflammatory mediators, secreted in response to insults, stimulate the participant cells and tissues to become or remain inflamed. In the absence of such stimulation, however, the cells and tissues passively recover from the inflamed state. In the early 2000's, this hypothesis began to be revised (Serhan, 2002). Resolution became to be viewed as an active, rather than a passive, process that is accelerated by endogenous pro-resolving agents. Accordingly, when the aim is to fight with pathologic inflammation, the promotion of pro-resolving processes can be hypothesized as an important goal as well as the suppression of the pro-inflammatory processes. To understand the details of this resolution paradigm on the cellular and molecular levels, the identities and functions of the pro-resolving molecules (hereafter referred to as pro-resolvents) have been intensely studied in recent years (Serhan et al., 2015). Among heterogeneous pro-resolving substances including peptides, nucleosides, steroids, and neurotransmitters, here we focus on fatty acid-derived ones. To date, about 30 pro-resolvents have been found in endogenous fatty acid pools in inflamed exudates. Most of these are metabolites of n−3 fatty acids, including E-series resolvins (RvEs), D-series resolvins (RvDs), maresins (MaRs), and neuroprotectin (NPD1).

Changes in cellular functions upon exposure to pro-resolvents are an important aspect of the resolution paradigm. A critical parameter driving these changes is ionic transport. Fluctuations in ion (e.g., Ca^2+^) concentrations can significantly affect intracellular signal transduction in many cell types. Moreover, ionic flux is essential for controlling neuronal excitability. Therefore, pro-resolvent interactions with cellular ion-permeating channels, if any, might be an important node for altering the inflammatory phase of cells. In fact, two axial mechanisms regarding such interactions have emerged. One involves ion channel modulation related to sensory neuronal excitability and the other involves that in mucus secreting pathways in the airway (Karp et al., 2004; Ji et al., 2011; Yoo et al., 2013; Lim et al., 2015). The molecular details of these two mechanisms have been investigated in the context of inflammatory pain sensation and cystic fibrosis, respectively. These studies have also suggested potential outcomes from the interactions between pro-resolvents and ion channels. Of these two mechanisms, we focus on the modulation of sensory neuronal ion channels and discuss further derived hypotheses for related disease progress and future perspectives.

## Fatty acid-derived pro-resolvents

Fatty acid metabolites are produced from immune cells including monocytes/macrophages, injured tissue, and the vascular endothelium during inflammation. After extracellular diffusion, these substances can be further processed biosynthetically to become final regulatory mediators which in turn interact with their partner receptor molecules. These interactions drive alterations in cellular function. These lipidergic components can be divided into two categories according to their regulatory directions in the inflammatory phases: pro-inflammatory and pro-resolving classes (Serhan et al., 2008; Ji et al., 2011). Although, a few exceptions exist, substances derived from n−6 fatty acids (e.g., prostaglandins and leukotrienes) generally serve pro-inflammatory roles. By contrast, pro-resolvents predominantly include n−3 derivatives.

Notable exceptions are lipoxin species, which are metabolites of n−6 arachidonic acid that exhibit pro-resolving activity. Among the lipidergic pro-resolvents, lipoxins were the first to have their chemical structures characterized and their biological actions identified (Serhan et al., 1984). Lipoxin was also the first lipidergic pro-resolvent to be studied in terms of an ion channel-associated action. In 1992, around which the curiosity regarding lipid-ion channel interaction was increasing due to the findings that some eicosanoids were shown to activate K^+^ channels (Buttner et al., 1989; Kim and Clapham, 1989), lipoxin A4 was demonstrated to excite dorsal root ganglionic sensory neurons as measured by surrogate outputs of increased bronchus contraction and neuropeptide secretion (Meini et al., 1992). Blockage and desensitization of the vanilloid subtype transient receptor ion channel (TRPV1) each prevented this effect, although it was not electrophysiologically confirmed, implicating that TRPV1 activity may be enhanced by lipoxin A4 exposure. Lipoxin A4 did not exhibit competitive binding with a TRPV1 agonist to the natural protein in the neuronal membrane. However, early binding assays were not perfectly reliable as it was later shown that TRPV1 protein was not actually used in this assay (Meini et al., 1992). Thus it remains elusive whether lipoxin A4 directly binds to this ion channel or indirectly modulates its activity via signal transduction events to exert this excitatory effect. It was considered unlikely that this n−6 derived lipoxin A4 resulted in neuronal excitation, which possibly conflicts with other known resolution mechanisms by which excitatory features are believed to be down-regulated. In contrast, n−3 metabolites, which constitute the majority of pro-resolvents, have commonly been shown to suppress TRP channel activities, eventually interfering with neuronal excitation, as described below.

Although, this review will not focus on this topic, the effects of lipoxins on ionic conductance have been studied more often in the context of airway fluid balance than in neuronal excitability (Karp et al., 2004). Lipoxins have been shown to promote the activity and/or expression of many different types of ion channels including epithelial Na^+^ channels, cystic fibrosis transmembrane conductance regulators, ATP-sensitive K^+^ channels, Ca^2+^-activated anion channels, pannexin 1 hemichannels, and canonical subtype of TRP channels (TRPCs). These effects lead to beneficial outcomes with respect to fluid secretion in the airway (Pang et al., 2011; Verriere et al., 2012; Buchanan et al., 2013; Wang et al., 2013; Yang et al., 2013; Al-Alawi et al., 2014; Higgins et al., 2014, 2015; Qi et al., 2015). Recent studies showing similarly beneficial effects from treatment of other n−3 type pro-resolvents have also emerged in the airway field (Hiram et al., 2014; Wang et al., 2014; Colby et al., 2016).

More than a decade after the discovery of lipoxins, n−3 type lipids began to be added in the list of pro-resolvents (Serhan et al., 2000). The n−3 fatty acid-derived pro-resolvents include D-series and E-series resolvins, their isomers, neuroprotectin D1, and maresins (Yoo et al., 2013). Variant intermediates are first produced from source lipids [e.g., n−3 docosahexaenoic acid (DHA) or eicosapentaenoic acid (EPA)] via pre-oxygenating processes by cytochrome P450, aspirin-triggered cyclooxygenase, or 12- or 15- lipoxygenases (LOXs). Next, 5-LOX generates the final forms of all these pro-resolvents. Each enzymatic step can occur in different types of participant cells including monocytes/macrophages, epithelial cells, and platelets, through intercellular diffusions of the intermediates, which is called transcellular biosynthesis (Yoo et al., 2013). Although, this process likely starts at the same time as the production of pro-inflammatory mediators such as various prostaglandins, the tissue levels of pro-resolvents seem to peak hours to days later, which may reflect the time needed for *de novo* transcription of the enzymes that produce pro-resolvents. Therefore, this time lag contributes to the duration of the inflammatory response.

The activation of specific G-protein coupled receptors (GPRs) and the resultant changes in cellular function explain the molecular mechanism for many of the actions of pro-resolvents on innate immune cells and inflamed tissues. For example, RvE1 (E designates the precursor lipid, EPA) activates the GPR, chemR23, and antagonizes the type 1 leukotriene B4 receptor (BLT1). RvD1 (D designates DHA) activates GPR32 and lipoxin A4/Annexin-A1 receptor/formyl-peptide receptor 2 (ALX/FPR2). RvD5 has also been shown to activate GPR32. RvD2 activates GPR18 (Lim et al., 2015). It is unclear whether other pro-resolvents also utilize GPR signaling. The cellular outcomes of these molecular actions are variable but commonly contribute to resolution: e.g., decreased production and secretion of pro-inflammatory mediators from immune cells and tissues, disturbance of recruitment and function of the polymorphonuclear neutrophils, increased recruitment of monocytes/macrophage; enhanced non-phlogistic phagocytosis of innate immune cells, and increased secretion of peptidergic pro-resolvents such as interleukin-10 from those cells (Yoo et al., 2013).

After accumulation of the efficacy indices and mechanistic information about the actions of pro-resolvents in the immunology field, scientific interest extended to include the relation with neuronal ion channels. This interest took 10 years to develop, longer than the period that it took from lipoxin finding to its historical approach targeting TRPV1 (Xu et al., 2010). It may be partly attributed to the limited availability of substances for the academic field to study. Most of the results have been produced from six pro-resolvents and four sensory neuronal TRP ion channels, which will be introduced, following a brief overview of the tradition of TRP channel-lipid interaction studies.

## TRP-lipid interactions

Mammals, including humans, express 28 or 29 TRP ion channel subtypes. Different from other six-transmembrane channels, most of which serve as voltage-gated channels, TRPs are only weakly sensitive to changes in the membrane voltage and play heterogeneous roles. In response to stimuli specific for each TRP, e.g., physical stimuli such as temperatures and mechanical forces, binding of second messengers, enzymatic modifications of the protein, and endogenous chemical activators, TRP channels open and allow predominantly Ca^2+^ and Na^+^ to permeate. This process leads to the depolarization of excitable cells or to the promotion of various Ca^2+^-mediated signaling processes in both excitable and non-excitable cells. With respect to the chemical binding, few cases of inhibitory modulation of TRP activity have been found but activation and potentiation constitute majority of reactions (Yoo et al., 2014).

Excluding the lipoxin experiment with native TRPV1 mentioned above owing to the lack of direct measurement of channel activity, the tradition of lipid interaction studies in the TRP field began in the late 1990s (Chyb et al., 1999; Zygmunt et al., 1999; Hwang et al., 2000). As soon as n−6 polyunsaturated fatty acids were reported to activate *Drosophila* TRP channels (Chyb et al., 1999), anandamide, hydroperoxyeicosatetraenoic acids (HPETEs), and leukotrienes, all of which are also n−6 fatty acid derivatives, were identified as TRPV1 activators (Zygmunt et al., 1999; Hwang et al., 2000) Since then, information on lipid activators has been expanded dramatically (Choi et al., 2014; Lim et al., 2015). The activities of TRPC and melastatin type TRP (TRPM) species are affected by the binding of second messengers such as diacylglycerol or its precursor, phosphatidylinositol 4,5-bisphosphate. Ankyrin type 1 TRP (TRPA1) is highly responsive to a broad range of lipid peroxides via a unique binding mode whereby peroxygenated lipids covalently bind to its cytosolic N-terminal nucleophilic amino acid residues. TRPVs tend to be activated by hydroperoxy- or epoxy-eicosanoids. Recent structural approaches have identified a putative lipid binding site in the intracellular regions of TRPV1 protein as described below. Most of those lipids can activate only their specific TRP channel and can also synergistically potentiate its activity in the presence of a stimulus with a different quality. These lipid activators are generated by various cells, even cells expressing TRPs. These molecules appear to behave in an autocrine and paracrine manner. TRP activations by these lipids in neurons mostly promote their excitability.

## Modulation of sensory TRP activities by pro-resolvents

Several TRPs serve as molecular sensors for environmental changes, expressed particularly highly in the skin-innervating terminals of somatosensory nerve C-fibers and Aδ fibers. Among 28 human subtypes, 6 members, which are TRPA1, TRPV1-4, and TRPM8, play this role and are collectively called sensory TRPs. These sensory TRP channels are all polymodal to some extent, which might be an evolutional strategy for enabling sensory neurons to monitor a variety of environmental changes. All 6 of these TRPs are responsive to a particular range of temperatures and 3 of them are sensitive to different qualities of mechanical stretching (Hwang and Oh, 2007). Regarding chemical sensitivities, a surprisingly large number of natural pungent substances, toxins, and different kinds of endogenous lipids, have been shown to activate sensory TRPs. In specialized sensory organs in the vision, olfaction, and gestation systems, the majority of sensor molecules are specific types of GPRs and TRP channels predominantly function as downstream effectors driving depolarization. In contrast, sensory TRPs seem to be the major receptors to detect environmental risks in the somatosensory system. This setup likely benefits from the high degree of polymodality of TRPs. Rather than with pleasant or explorative quality, perception by our brain resulting from TRP activation is with a nociceptive one: pain. This attribute frequently places TRP modulation as a desirable goal at the viewpoint of analgesia which can be rephrased as resolution of pain (Lim et al., 2015). Many studies of the interactions of TRPs with fatty acid-derived pro-resolvents have adhered to this angle. Six pro-resolvents have been investigated in terms of their actions on five different neuronal ion channels, four of which are TRPs. All the pro-resolvents negatively modulated the ion channel activities, indicating that these lipids are potential analgesics (Table 1).

**Table 1:** **List of neuronal ion channels that are modulated by lipidergic pro-resolvents**.

**Ion channel**	**Lipidergic pro-resolvents**
TRPA1	Resolvin D1 (Bang et al., 2010), Resolvin D2 (Park et al., 2011b), Maresin 1 (Serhan et al., 2012)
TRPV1	Resolvin D2 (Park et al., 2011b), Resolvin E1 (Xu et al., 2010; Park et al., 2011b), Neuroprotectin D1 (Park et al., 2011a), Maresin 1 (Serhan et al., 2012; Park, 2015)
TRPV3	AT-resolvin D1 (Bang et al., 2012a), Resolvin D1 (Bang et al., 2010)
TRPV4	Resolvin D1 (Bang et al., 2010)
NMDA receptor	Resolvin E1 (Xu et al., 2010), Resolvin D1 (Quan-Xin et al., 2012)

### TRPA1 modulations by D-series resolvins and MaR1

As detailed above, the TRPA1 ion channel is expressed in only a subpopulation of pain-mediating nociceptors. Some types of physical and chemical insults that are potentially harmful to the body can affect the structure of TRPA1 protein, leading to allosteric opening of this channel. Thus TRPA1 is often called a “damage-sensing ion channel.” TRPA1 is particularly sensitive to noxiously cold temperatures (<17°C; Story et al., 2003; Obata et al., 2005; Kwan et al., 2006; Karashima et al., 2009; Del Camino et al., 2010; Chen et al., 2011), harsh mechanical deformations (Petrus et al., 2007; Kerstein et al., 2009), and the binding of environmental or endogenously generated toxic substances including diverse lipid peroxides, reactive chemicals, and pollutants (Bang and Hwang, 2009; Kim and Hwang, 2013). In response to these stimuli, TRPA1 opens and depolarizes the nociceptors. Hardly shown in activation mechanisms for other ion channels, covalent reactions with specific nucleophilic amino acids in the cytosolic N-terminus of TRPA1 with α,β-unsaturated carbon-containing substances seem to underlie the ligand-mediated gating of TRPA1 (Hinman et al., 2006; Macpherson et al., 2007; Andersson et al., 2008; Bang and Hwang, 2009). In addition, TRPA1 also acts as a final effector in intracellular signal transduction for inflammatory mediators such as nerve growth factor (NGF) and bradykinin, indicating that TRPA1 is critical for inflammatory pain sensation (Bandell et al., 2004; Bautista et al., 2006; Kwan et al., 2006; Petrus et al., 2007; Cenac, 2013). Due to this polymodality and the distinctively limited number of TRPA1-positive neurons, these neurons are frequently subcategorized into polymodal nociceptors. The identification of a mechanism for inhibiting TRPA1 activity, would greatly contribute to the resolution of pain states (Baraldi et al., 2010).

Of particular note, pro-resolvents seem to have a central role for endogenous inhibition of TRPA1. For instance, resolvin D1 (RvD1) strongly blocks TRPA1 activity (Bang et al., 2010). This channel blocking potency indeed extrapolates to the *in vivo* level by showing that TRPA1 agonist-induced chemical pain were attenuated by RvD1 administration. TRPA1 also takes part in the mechanosensory mechanisms, and RvD1 was shown to prevent mechanical hypersenstivity in complete Freund's adjuvant (CFA)-inflamed animals. Since RvD1 is locally confined when injected and outcomes were measured at relatively acute time points, the pain relief observed *in vivo* appears to be mediated in the absence of intercellular actions such as recruitment or activation of innate immune cells.

**Figure 1:**
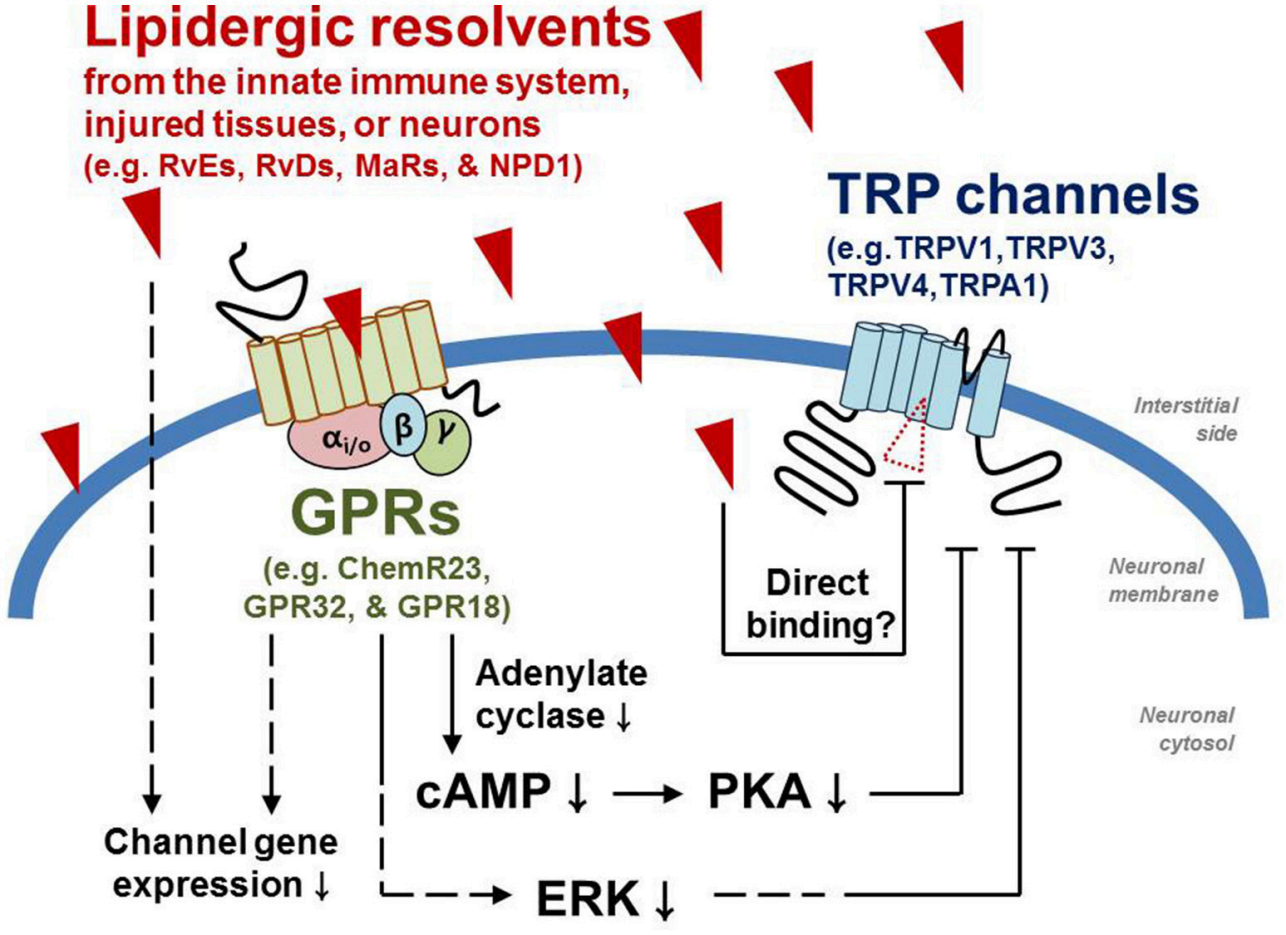
**Molecular modes of action by which pro-resolvents inhibit TRP channels**. Dotted lines indicate that other unknown signaling components may take part during the pathways.

RvD2 also inhibits TRPA1 activity (Park et al., 2011b). G-protein-mediated signal transduction seems to intervene the molecular mechanism of this process while such an explanation is unlikely for RvD1: the inhibitory action of RvD2 was shown to be prevented by the uncouplers for Gαi-cycling, pertussis toxin, and GDPβS. The specific GPR that initiates the signal has yet to be identified. On the other hand, specific agonists for ALX/FPR2, the GPR on which RvD1 acts, such as cathelicidin LL-37 and Trp-Lys-Tyr-Met-Val-Met (WKYMVM) are inert to TRPA1 activation or inhibition (Bang et al., 2010). To confirm their mechanism of action, the different modes for these related substances for the same target protein may need to be revisited (Figure 1). Subtle structural difference (e.g., differences in the location of one hydroxyl moiety and in the cis-trans nature of carbon links) may determine the receptor binding specificity. With respect to behavioral outcomes, no significant discrepancy was observed. TRPA1-mediated mechanical phenotype was greatly improved under inflamed conditions (Park et al., 2011b).

MaR1 is a recently identified pro-resolvent that has been demonstrated to elicit somewhat contradictory outcomes. *In vitro* electrophysiological measurements indicated that TRPA1 activity is not affected by the presence of MaR1 (Serhan et al., 2012). However, vincristine-induced *in vivo* mechanical hypersensitivity, to which TRPA1 is known to contribute, was significantly alleviated by MaR1 treatment. Without direct TRPA1 involvement, MaR1 may act downstream or upstream of TRPA1 mediation in pathological processes.

### TRPV1: an important node for various resolving actions

With a greater polymodality than that of TRPA1, TRPV1 serves as a central pain receptor in the body. Potentially damaging heat (>42°C), acidosis, abnormal hypertonicity, endogenous lipidergic pro-inflammatory messengers such as leukotrienes, and natural pungent compounds such as capsaicin and tarantula toxin evoke pain via direct structural challenges to TRPV1 protein, culminating in its electrical activation (Caterina et al., 1997, 2000; Tominaga et al., 1998; Davis et al., 2000; Hwang et al., 2000; Siemens et al., 2006; Vriens et al., 2009; Nishihara et al., 2011). Furthermore, major pro-inflammatory mediators such as bradykinin, prostaglandin, and NGF utilize TRPV1 as their downstream effector, thereby causing pathologic exacerbation of pain and inflammation (Basbaum et al., 2009). These findings have implicated TRPV1 as the forefront node between painful insult and human and mammalian perception of$ it (Hwang and Oh, 2007). While such nodal actions are well-defined in the periphery of the sensory neurons, evidence is currently expanding for its contribution in the central terminals and even in spinal interneurons to the boosting pain circuit, which suggests that its different nodal roles exist in the central synapses (Choi et al., 2016). Therefore, TRPV1 may also be a critical pain resolving node because the extensive scale of pain pathologies could be controlled if TRPV1 activity could be tuned.

In the same way for TRPA1 inhibition, RvD2 suppresses TRPV1 activity. Through unknown GPR activation by RvD2, intracellular Gαi-coupled signaling leads to decreased TRPV1 activity. Of all the pro-resolvents whose ability to inhibit TRP has been studied, RvD2 exhibits the strongest potency (IC50 ~100 pM). Predictably, acute pain induced by capsaicin and inflammatory heat hyperalgesia, which are the *in vivo* modalities that TRPV1 predominantly covers, were both blunted by RvD2 treatment (Park et al., 2011b).

Similarly, RvE1 and MaR1 have been found to inhibit TRPV1 activity and TRPV1-mediated pain behavior, but did not affect modalities involving TRPA1 (Xu et al., 2010; Park et al., 2011b; Serhan et al., 2012; Park, 2015). Since RvE1 acts on a single GPR (chemR23), it can be hypothesized that certain GPR signaling pathways may be more tightly coupled to specific TRP subtypes. In fact, coexpression of chemR23 and TRPV1 has been confirmed in a subset of nociceptors (Xu et al., 2010).

Neuroprotectin D1 (also called protectin D1 or NPD1), another D-series pro-resolving lipid, has also been shown to inhibit TRPV1 albeit one third of the potency of RvD2 (Park et al., 2011a). A Gαi protein-coupled indirect mechanism was again suggested to underlie this inhibitory action, although the specific GPR involved is not known. Other signaling components that can amplify TRPV1activity may also be affected by NPD1, for example, protein kinase A (PKA) and extracellular signal–regulated kinase (ERK; Park et al., 2011a). Moreover, TNF-α-mediated spinal synaptic transmission was attenuated and the associated behavioral phenotypes were extrapolated in a manner partly dependent on TRPV1 inhibition. Recently, NPD1 treatment also exhibited beneficial effects on neuropathic pain in an animal study (Xu et al., 2013).

### TRPV3 modulation by D-series resolvins

Several aspects may make one hesitate to conclude that TRPV3 is a nociceptive component. For example, TRPV3 begins to open in response to relatively mild heat radiation that results in innocuously warm temperatures (>33°C), whereas the temperature threshold for TRPV1 activation is close to the range reported to actually cause pain perception *in vivo* (Caterina et al., 2000; Moqrich et al., 2005). The main expresser cells are not sensory neurons but non-excitable basal keratinocytes in the epidermis (Peier et al., 2002). The presence of TRPV1 seems to be sufficient to monitor external noxious temperatures (Peier et al., 2002; Smith et al., 2002; Xu et al., 2002, 2006). Although, the precise intercellular circuitry involved in TRPV3-mediated somatosensory signaling has yet to be fully unraveled, pharmacological, and genetic studies have yielded some insights. Industrial drug developments targeting TRPV3 have shown that synthetic TRPV3 blockers commonly exhibit painkilling efficacy in inflammatory and neuropathic pain (Facer et al., 2007; Bevan et al., 2008; Broad et al., 2009; Khairatkar Joshi et al., 2010; Reilly and Kym, 2011). More importantly, TRPV3-knockout animals have shown significant tolerance to painful heat (Moqrich et al., 2005; Huang et al., 2008; Miyamoto et al., 2011). Thus, TRPV3 participates in nociception of noxiously high temperatures albeit to a lesser extent than TRPV1, and keratinocytes may contribute to thermosensation possibly by transmitting their depolarization signals to neighboring nociceptor neurons.

Consistently, both resolvins that modulate TRPV3 activity exert painkilling effects. Aspirin-triggered RvD1 (AT-RvD1 or 17(R)-RvD1) has been shown to specifically block TRPV3. The *in vivo* administration of this compound has been shown to mitigate heat pain, a TRPV3-mediated sensory modality, but not mechanical pain under inflammatory conditions (Bang et al., 2012a). RvD1, which is relatively promiscuous in TRP interactions (as mentioned for TRPA1 above and TRPV4 below), has also been shown to inhibit TRPV3 channel opening and TRPV3-mediated pain (Bang et al., 2010). As shown in TRPA1 findings, AT-RvD1 and RvD1 unlikely utilize G-protein-coupled signaling for TRPV3 inhibition because these effects were resistant to treatment of the Gβγ inhibitor gallein and to treatment of the GPR agonists LL37 and WKYMVM (Bang et al., 2010, 2012a). Other indirect mechanisms such as participation of innate immune cells were also excluded by localizing RvDs to limited regions and times and showing that the effective doses were different than those for leukocytes (Krishnamoorthy et al., 2010).

### TRPV4 modulations by RvD1

Albeit to a lesser extent than TRPV1 and TRPA1, TRPV4 is also a polymodal sensor ion channel. Elevated temperatures (>27–34°C), mechanical stretching caused by pressure or swelling, and endogenous pro-inflammatory mediators such as epoxyeicosatrienoic acids (EETs) and dimethylallyl pyrophosphate are able to activate TRPV4 (Liedtke et al., 2000; Strotmann et al., 2000; Watanabe et al., 2003; Levine and Alessandri-Haber, 2007; Bang et al., 2012b). This activation is believed to lead to depolarization of the primary nociceptors, ultimately resulting in pain perception via the ascending transmission of this signal. While changes in thermonociceptive behaviors affected by TRPV4 knockout were significant but limited compared to phenotypes reported from genetic ablation of TRPV1 or TRPV3 (Todaka et al., 2004; Lee et al., 2005), the mechanosensory modalities that involve TRPV4 appear to be remarkable. Many types of inflammation caused somatosensory mechanical hyperalgesia or visceral mechanical hyperalgesia via TRPV4 activation (Alessandri-Haber et al., 2006; Chen et al., 2007; Grant et al., 2007; Brierley et al., 2008; Cenac et al., 2008). In the context of GPR-mediated inflammatory pain signaling, activation of protease-activated receptor 2 (PAR2, also known as GPR11) by trypsin or thrombin secreted from tissues is linked to TRPV4, which functions as its downstream effector for nociceptor neuronal firing (Grant et al., 2007). Histamine and serotonin also seem to contribute to GPR cascade, culminating in TRPV4 potentiation, thereby exacerbating visceral pain (Brierley et al., 2008; Cenac et al., 2008, 2010). In two different models of neuropathic pain, a chronic constriction injury model and a taxol-induced neuropathy model, TRPV4 contributed to increased mechanical pain (Alessandri-Haber et al., 2004; Zhang et al., 2008).

RvD1 is the only resolvin to date found to inhibit TRPV4. The Levine lab established an *in vivo* method for indexing TRPV4-specific somatosensory mechanical pain phenotypes. In this approach, hypotonic stimulation is applied locally to a rat paw when the paw is pre-primed with an inflammatory mediator prostaglandin E2 (Alessandri-Haber et al., 2003, 2006; Chen et al., 2007). The Hwang lab has reproduced this model with mice (Bang et al., 2010, 2012b). RvD1 was shown to be effective at alleviating TRPV4-specific inflammatory mechanical pain (Bang et al., 2010). Heat hyperalgesia in CFA-induced inflammation was also blunted by *in vivo* treatment of RvD1. Of the four heat sensor TRPs (TRPV1-4), TRPV3 and TRPV4 have been identified as molecular targets of RvD1. However, it remains unclear whether TRPV3 or TRPV4 predominates over the RvD1 effect on the thermal phenotype.

Using the same experimental approach described above for TRPA1 and TRPV3, an indirect ALX/FPR2-mediated GPR pathway has been excluded from the potential molecular interacting mechanism underlying these results. Although, it is unclear without binding assay, modulation via direct contact can be considered from what was recently hypothesized. It is suggested that chemical interactions between the lipid components of the bilayer membrane and the embedded regions of ion channel proteins are important for appropriate ion channel response and gating (Bavi et al., 2016). For ion channels with six transmembrane domains (which include TRP channel), one of the most critical regions in this regard is the intracellular linker between transmembrane domain (TM) 4 and TM5. For voltage-gated channels, this linker is crucial for allosteric conversion of movement of the TM4 voltage sensor upon depolarizing stimulation into gating of the pore located between TM5 and TM6. According to recent structural approaches, the same linker of TRPV1 protein seems to bind to a lipidergic component and may also be responsible for transducing this binding energy to the pore region (Cao et al., 2013; Liao et al., 2013). The corresponding region of a bacterial mechanosensitive ion channel has been shown to be important since membrane stretching appears to alter binding between the linker and the bilayer lipids, resulting in pore opening (Bavi et al., 2016). Therefore, it is hypothesizable that the structure of this linker region is vulnerable (or sensitive) to external physical or chemical challenges (e.g., voltage increases that outwardly tilt the TM4 helix, membrane stretching that dysregulates bilayer contacts, lipidergic ligands that substitute a bilayer component, etc.), subsequently affecting the condition of the pore. In its closed states the linker region of TRPV1is pre-occupied by an endogenous lipid that has not yet been identified (Cao et al., 2013). In the structure of TRPV4, the closest homolog of TRPV1 (Teng et al., 2015), the lipid activators such as EETs may compete with the pre-occupying lipid, as predicted from capsaicin or HPETE interaction with TRPV1 (Hwang et al., 2000; Jordt and Julius, 2002). Conversely, RvD1 may more strongly stabilize the resting state than the pre-occupying endogenous one when located in the same place (Figure 1). When previous observations of lipidergic activators are considered together, it is possible that different double-bond locations of n−3 and n−6 derivatives may first direct stabilizing and destabilizing effects, and that the carbon lengths and locations of hydroxyl moieties that likely determine optimal occupation and hydrogen bond potential may define TRP specificity (Chyb et al., 1999; Hwang et al., 2000; Hu et al., 2006; Andersson et al., 2007; Komatsu et al., 2012; Motter and Ahern, 2012). Future experimental evidence is necessary to prove this hypothesis.

### N-Methyl-D-aspartic acid (NMDA) receptor modulation by resolvins

Nociceptor populations among primary sensory neurons propagate peripheral pathologic signals, including those originating from inflammation, to the central nervous system. Moreover, these neurons actively contribute to pro-inflammatory processes via neurogenic inflammation mechanisms, by interactively exchanging various intercellular mediators with innervating tissues. These are important steps in the development of chronic inflammatory pain. In terms of pain relief, the TRPs mentioned above are essentially focused on when resolution mechanisms are examined, since the activation of these ion channels explains the outset of nociceptor excitation and pathologic hyperactivity, which often exacerbate neurogenic inflammation. The actions of pro-resolving lipids through these sensory TRP modulations all seem to converge on pain alleviation.

Postsynaptic dorsal horn neurons translate and relay signals received from the synaptic inputs of the central terminals of nociceptor neurons in the spinal cord (Choi et al., 2016). This synaptic transmission is the next gate to the development of persistent pain, which is determined by whether this spinal synapse is strengthened by a barrage of peripheral input. Postsynaptic NMDA receptor ion channels play a key role in this process (Lim et al., 2015). Pro-resolvents also down-regulate postsynaptic NMDA function in this central postsynapse, but partly in a different manner than for TRP channel modulations. The same GPR, chemR23, is involved in RvE1 action, but the dorsal horn-specific ERK phosphorylation cascade, which promotes NMDA potentiation, was mainly suppressed (Xu et al., 2010). A later RvD1 experiment has demonstrated another aspect of functional down-regulation of NMDA by showing that RvD1 treatment decreased phosphorylation of two NMDA subunits, NR1 and NR2B, in a model of pancreatitis-induced allodynia, but without confirming the knowledge of related GPRs (Quan-Xin et al., 2012).

Upon RvE1 exposure, presynaptic ERK phosphorylation has also been shown to be suppressed. In addition, the frequency of spontaneous excitatory postsynaptic potentials, which reflects not only postsynaptic function but also the extent of presynaptic glutamate release, was also decreased. This finding may also explain the RvE1-mediated suppression of TRPV1-independent presynaptic TNF-α signals (Xu et al., 2010). The mechanistic difference that occurs in the central synaptic region is conceivable while TRP channel modulation by pro-resolvents, which is probably more important in the periphery, is presumed to be mediated via direct binding or a rapid signal transduction through tightly coupled G-protein cycling.

## Perspectives and conclusions

Here we reviewed the effects of various pro-resolving lipids on the functions of four sensory TRP channels and NMDA receptors that convey nociceptive signals. These pro-resolvents commonly showed strong inhibitory actions on these ion channels, leading to a final outcome of pain resolution. It is perhaps surprising that a group of endogenous lipid derivatives from common precursors can contribute to the resolution of two different pathologic processes, pain and inflammation, via differential cellular targets and biological mechanisms. Immunology studies continue to confirm that the absence of adverse effects from *in vivo* treatment of these substances. Based on current efficacy and safety information, translational applications of these substances to some specific diseases are eagerly anticipated. Before this promise can be realized, it would be advantageous to establish proof of concept to clarify the relevant molecular mechanisms. The most ambiguous ones to date are the binding mode by which these pro-resolving lipids interact with channel proteins and the mechanisms how they initiate GPR-mediated signal transduction. Although, one putative lipid-interacting site has been predicted, it has not yet been established whether resolvins modify channel activity through this site. When GPR coupled, it has not been completely answered which GPR predominantly drives the effect and which intracellular signal deactivates the channel. Most studies have focused on the sensory circuit. TRPV channels are involved in plastic changes in other brain synapses (Shibasaki et al., 2007; Gibson et al., 2008). NMDA receptors are essential ion channels for synaptic strengthening throughout the whole brain. In addition to ion channel interaction in excitable neurons, changes in microglial function similar to those in monocyte/macrophage function may also be important checkpoints as once shown (Xu et al., 2010). Pro-resolvents are endogenously generated. Although, neuronal ion channels are strongly regulated by extraneous treatment, it remains to be elucidated which the major sources are among components in the nervous system and how similarly their transcellular biosyntheses occur as previously reported in the immune system.

Lessons from studies of the airway or other tissues may help to frame a different paradigm. In the airway research field, rather than channel activation or inhibition, changes in the expression of an ion channel are a more important axis to define the effect of a pro-resolvent, which have received little attention in the neuroscience field. The activity of Ca^2+^-activated anion channels is increased upon pro-resolvent treatment in the airway epithelium (Verriere et al., 2012). These channels are considered to be an important temperature sensor and depolarizer in nociceptor neurons (Cho et al., 2012). However, this interaction has yet to be tested for neuronal outcomes. Future studies for all these questions will help determine whether these pro-resolvents (or future synthetic analogs thereof) will have clinical applications, how firmly the proof of concept needs to be established, and which pieces are missing in order to fully explain the molecular puzzle of ion channel interactions with lipidergic pro-resolvents.

## Author contributions

GC analyzed documented results and prepared the preliminary draft. SH supervised the study and finalized the manuscript. All authors read and approved the final manuscript.

### Conflict of interest statement

The authors declare that the research was conducted in the absence of any commercial or financial relationships that could be construed as a potential conflict of interest.
